# Promoting Achievement of Level 1 Milestones for Medical Students Going into Emergency Medicine

**DOI:** 10.5811/westjem.2016.10.31247

**Published:** 2016-12-05

**Authors:** Cynthia G. Leung, Laura Thompson, Jennifer W. McCallister, David P. Way, Nicholas E. Kman

**Affiliations:** * Ohio State University College of Medicine, Department of Emergency Medicine, Columbus, Ohio; † Ohio State University College of Medicine, Department of Internal Medicine, Columbus, Ohio

## BACKGROUND

Over the past decade, U.S. medical schools have begun reformulating their fourth-year curricula, moving from an open format of career exploration and audition electives to a more structured program designed to prepare students for patient-care responsibilities upon entering residency. 1–4 This trend is attributable to recommendations handed down from several key organizations. In 2011, the Alliance for Clinical Education (ACE) endorsed the use of the Accreditation Council of Graduate Medical Education (ACGME) Core Competencies and the Association of American Medical Colleges (AAMC) Entrustable Professional Activities (EPA) to guide medical educators in redesigning the fourth-year curriculum. Additionally, ACE published four specific guidelines. First, they recommended that all students demonstrate progress towards mastery of the six ACGME Core Competencies. Second, they stated that all students should complete a capstone course specifically designed to prepare them for residency. Third, they said that medical school curricula should provide specialty-specific objectives to prepare students for residency in their intended specialty. Finally, they endorsed a system for helping students identify and correct gaps in their knowledge and skills during the fourth year. 1

The authors began their efforts to respond to the call for fourth-year curriculum revision with a review of the literature, specifically looking for what medical graduates entering emergency medicine (EM) were lacking upon entry into residency. A study by Lyss-Lerman found that program directors believed that interns’ primary shortcomings were in the areas of medical knowledge, professionalism, organizational skills, and self-reflection.^4^ More recently, the development of Level 1 ACGME Milestones has helped to more clearly articulate expectations of graduating medical students upon entering residency.^5^ Weizberg, et al. conducted a multi-institution study in which EM interns were assessed on eight Level 1 milestones within the first month of residency and found that fewer than 75% met Level 1 for any of the eight milestones assessed.^6^ These studies emphasize the need to revise curricula to better prepare students for the transition from undergraduate to graduate medical education.

To bridge the gap between traditional third-year core clerkships and the internship year, our institution introduced courses of study customized for our student’s intended specialty. The courses of study, called “Clinical Tracks,” are longitudinal across the fourth year of medical school and are designed to prepare students for the next stage of training by offering a framework for entry level, specialty-specific learning milestones. The EM Clinical Track, with associated learning objectives and assessments, was based on the EM milestones developed jointly by the ACGME and the American Board of Emergency Medicine (ABEM).^7^

## OBJECTIVES

The objective of this innovation was to transform a traditional fourth year-curriculum into a program designed to better prepare medical students for a residency in EM. The result was the Clinical Track in EM, a competency-based curriculum that offered medical students the opportunity to achieve and demonstrate competency in all 23 of the Level 1 EM milestones.

## CURRICULUM DESIGN

The Clinical Track in EM was conceived as a comprehensive longitudinal curriculum comprised of a series of required fourth-year clerkships supplemented with a menu of recommended electives. With guidance from a faculty advisor, the students designed a clinical track that provided them with the best opportunities to develop knowledge and skills deemed essential for starting an internship in EM. A critical feature of the EM clinical track was a series of competency-based assessments designed to provide students with feedback on their progress toward achieving Level 1 EM milestones.

Pre-existing learning objectives from the required fourth-year clerkships, including clerkships in EM, ambulatory medicine, chronic care, and intensive care, were mapped to the Level 1 EM milestones. Many of the pre-existing assessments within these clerkships were determined to provide the information needed to assign student performance levels for most of the 23 EM milestones. The pre-existing assessments included the following:

1. EM clerkship Clinical Performance Assessment (CPA) provided a global assessment of patient care milestones based on end-of-shift performance evaluations gathered from numerous faculty over the course of the clerkship.
2. EM clerkship EPA 10 simulation assessment provided a standardized measure of a student’s management of the emergent patient in a realistic emergency department (ED) setting.^8^
3. EM clerkship procedure-lab assessments measured Level 1 milestones for airway management, ultrasound, wound care and vascular access.
4. EM clerkship quizzes measured core medical knowledge and the application of knowledge to clinical problems.
5. The ambulatory medicine clerkship Critical Appraisal of Topic (CAT) assignment was used to measure how well a student used evidence-based medicine to appraise a clinical question.
6. The Health Systems, Informatics and Quality (HSIQ) project, a longitudinal experience in which students identified a system failure in care delivery and wrote a proposal for a viable quality improvement intervention, assessed understanding of healthcare delivery systems.^9^

To fill the gaps, new assessments specifically for the Clinical Track in EM were developed and incorporated into a clinical elective called Advanced Topics in Emergency Medicine (ATEM).^10^ The new assessments included the following:

1. Assessment shifts in which students were evaluated on specific EM milestones through direct observation of a patient encounter by core education faculty. A key feature of the assessment shift is the observation instrument, which contains behavioral anchors taken directly from the Level 1 and 2 EM milestones. This facilitated the assignment of milestone levels. A copy of the instrument used for assessment shifts is included as an appendix; faculty completed this form on a tablet device, using the MyProgress Software Platform.^11^ ATEM students were required to complete three assessment shifts.
2. A capstone simulation assessment based on the EM oral board’s triple case^12^ was designed to assess EM milestones that are more difficult to evaluate in the clinical setting such as emergency stabilization (PC1) and multitasking (PC8).
3. A procedure log for logging procedures performed in the clinical environment, and checklist assessments for evaluating procedures performed in simulation.
4. A patient follow-up log required students to review cases seen in the ED, identify members of the care team and delineate the resources involved in the patient’s care.
5. Additional knowledge quizzes specific to Level 1 EM milestones were also added to the ATEM course.

A clinical competency committee (CCC) consisting of the clinical track director, the EM clerkship director, and the Part 3 (fourth-year) director reviewed the relevant assessment data for each student. Using the ACGME-ABEM scoring rubric,^7^ each student was assigned a level for each of the 23 EM milestones. Level assignments were based on a student’s consistent performance at not only the assigned level, but lower levels as well. For instance, assignment to a Level 2 required performance of both Level 1 and Level 2 criteria for any given milestone. An intermediate level (i.e. 1.5) was assigned if a student demonstrated only some of the higher level behaviors. Multiple sources of assessment data were used to assign levels for each milestone with the exception of a few of the procedural and systems-based milestones as shown in Table 1. In cases that contained conflicting assessment data, the most recent evidence was used, particularly if the student showed improvement over time.

## IMPACT/EFFECTIVENESS

### Outcomes

Seventeen students from a class of 185 enrolled in and completed the Clinical Track in EM during the inaugural year. The assessment data gathered throughout the clinical track year was sufficient for the CCC to assign a milestone level for students on 21 of the 23 EM milestones. Most students attained Level 1 or higher for 17 of the 23 EM milestones (see Table 2, and Figure). Notable exceptions include PC5-*Pharmacotherapy* (most students failed to consistently ask about allergies to medications); PC14-*Vascular Access* (a little more than 35% failed to perform arterial puncture); and PROF2-*Accountability* (more than half of the students failed to turn in their patient follow-up logs). The figure shows the median scores of the 17 students (boxes) and the range of scores (whiskers) for each of the 23 milestones. The Ohio State University Institutional Review Board determined this evaluation to be exempt from review.

The CCC panel was unable to fully assess two milestones, PC11-*Anesthesia/Acute Pain Management* and SBP2-*Systems-based Management*. For PC11, the panel assigned all students a Level 0.5 once it was discovered that students were never formally assessed on contraindications and complications of local anesthesia, a critical part of this milestone. Like PROF2, SBP-2 was affected by the failure of students to complete the required patient follow-up log assignment, which was also used to assess this milestone.

## DISCUSSION

The Clinical Track in EM was designed to transition the traditional fourth-year medical school curriculum to one based on competencies defined by the ACGME and the EM milestones. This transition required more structure and more formal assessments than existed in the traditional course of study. The Clinical Track in EM relied on assessment data gathered longitudinally throughout the fourth year of medical school. However, the data provided integrated and comprehensive information sufficient for assigning EM milestone performance levels.

One of the strengths of this program involved numerous observations of student performance by multiple faculty evaluators. The information gathered in this manner helped to capture some of the contextual variability inherent in the more complex patient care-based milestones.^13^ Additionally, due to the longitudinal nature, faculty were able to document student growth over time.

Another strength was that most evaluations were performed in realistic settings, either the actual or simulated ED. These assessment settings lend increased authenticity to the clinical performance assessments. Finally, milestone-level determinations were made by consensus of a CCC in undergraduate medical education made up of faculty who were thoroughly involved with the clinical track students throughout the fourth year. Their familiarity with the students contributed to confident decisions about the student’s milestone-level assignments.

## LIMITATIONS

The authors experienced several challenges in implementing the Clinical Track in EM. First, due to a technical problem with the electronic recording system adopted for entering assessment shift observations,^11^ 32% of the assessment shift data was lost, affecting 10 of 17 students. Fortunately, because multiple observations and multiple methods of assessment were used, milestones were able to be assigned, even with missing data. In the future, however, the authors recommend a rigorous trial period for any software program used to gather high-stakes evaluation information. Additionally, some patient care-based milestones, such as emergency stabilization (PC1) and multitasking (PC8) as well as many of the procedural milestones, could not be evaluated in the ED due to a shortage of appropriate patient encounters. Medical students are lowest on the hierarchy for such opportunities, so these competencies had to be assessed solely through simulation. Finally, the authors found it challenging to assess the systems-based practice and the practice-based performance improvement competencies in either an actual or simulated ED. As a result, they relied on information from other fourth-year curriculum projects and assessments. Although these activities were not carried out in the ED, the goals and objectives of these assessments were well aligned with the goal to provide competency-based assessment of EM milestones.

Much of the assessment of medical students relies heavily on the direct observation that occurs on the assessment shifts. The program leaders at our institution were able to accomplish this due to the preexistence of required teaching shifts by core faculty (of our residency program). Assessment shifts might be difficult to achieve for medical schools where teaching shifts are not feasible.

In preparation for the next academic year, the authors have already incorporated changes to the clinical track curriculum to improve our ability to assess and assign students to levels on the EM milestones. Changes include improvements to the pharmacotherapy curriculum to incorporate formal assessment of the student’s competency in applying knowledge of contraindications and complications in cases in which local anesthesia is required. A fully functional electronic assessment system for recording and saving direct observation data has been tested and implemented. And finally, the program leaders are implementing a “feed-forward” process to residency directors modeled after that described by Sozener 14 so that residency programs can make practical use of our efforts to document student performance during medical school.

## CONCLUSION

The goal of the Clinical Track in EM was to contribute to a continuity of education, bridging the continuum of medical education from medical school through residency and on into early practice. Communication of progress and achievement through the milestone structure can contribute to establishing this continuity of education. Compared to the observations by Weizberg, et al. (who found that fewer than 75% of EM interns had achieved Level 1 on the eight patient care-based milestones assessed upon entry into residency), almost all of our graduates achieved at least a Level 1 designation for 20 of 23 milestones. The creation of a specialty-specific EM clinical track provided the structure necessary to prepare medical students for their intended specialty. Key to this program was efforts to customize assessments to measure the ACGME EM milestones. Eventually, this assessment data will certify the graduating medical student’s preparation to begin an EM residency program.

## Supplementary Information



## Figures and Tables

**Figure:**
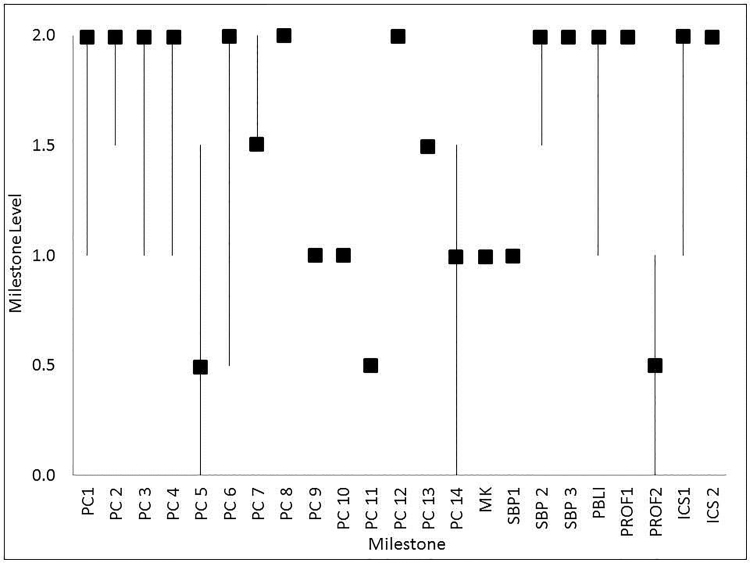
Median plot of milestone levels attained by 17 medical students who participated in a longitudinal emergency medicine clinical track (a series of required clerkships and electives) during their fourth year of medical school.

**Table 1:** Methods of assessment in a milestone-based Clinical Track in Emergency Medicine, as part of a fourth-year medical school curriculum.

Milestone	EM clerkship				Clinical track assessments				Other required experiences
	
	CPA	Quiz	Simulation	Procedure checklist	Assessment shifts	Quiz	Procedure logs	End of year simulation	
Emergency stabilization			x		x			x	
History and physical	x				x			x	
Diagnostic studies					x			x	
Differential diagnosis					x			x	
Pharmacotherapy					x	x		x	
Reassessment					x			x	
Disposition					x			x	
Multi-tasking					x			x	
Procedures				x			x		
Airway				x			x		
Anesthesia/pain								x	
Ultrasound		x		x			x		
Wound care				x			x	x	
Vascular access				x			x		
Medical knowledge									*
Patient safety									†
Systems management					x				‡
Technology					x				
PBLI					x				§
Professional values					x				
Accountability					x				
Patient communication					x				
Team management					x				

*PBLI*, practice-based learning and improvement; *CPA*, clinical performance assessment.

* Passing score on USMLE and EM Advanced Clinical Exam.

† Computer Based Learning Modules.

‡ Health Systems, Informatics and Quality Assignment (HSIQ); Patient follow up log.

§ Critical Appraisal of Topic assignment; Patient follow up log.

**Table 2:** Number and percentage (in parentheses) of 17 emergency medicine clinical track students by milestone level attained prior to graduation from medical school. Students in the missing category had incomplete information from assessments during the fourth year.

		Milestone level				
			
	Milestones	< 1	1	1.5	2	Missing
PC1	Emergency stabilization		1 (5.9)	4 (23.5)	12 (70.6)	
PC 2	Focused history & physical			2 (11.8)	15 (88.2)	
PC 3	Diagnostic studies		2 (11.8)	2 (11.8)	13 (76.5)	
PC 4	Diagnosis		1 (5.9)	4 (23.5)	12 (70.6)	
PC 5	Pharmacotherapy	14 (82.4)		3 (17.6)		
PC 6	Observation & reassessment	2 (11.8)	3 (17.6)	1 (5.9)	11 (64.7)	
PC 7	Disposition			12 (70.6)	5 (29.4)	
PC 8	Multi-tasking				17 (100)	
PC 9	Procedures		17 (100)			
PC 10	Airway management		17 (100)			
PC 11	Anesthesia/acute pain management	17 (100)				
PC 12	Ultrasound				17 (100)	
PC 13	Wound care			17 (100)		
PC 14	Vascular access	6 (35.3)	3 (17.6)	8 (47.1)		
MK			17 (100)			
SBP1	Patient safety		17 (100)			
SBP 2	Systems-based management			4 (23.5)	4 (23.5)	9 (52.8)
SBP 3	Technology		16 (94.1)			1 (5.9)
PBLI			8 (47.1)		9 (52.9)	
PROF1	Professional values				16 (94.1)	1 (5.9)
PROF2	Accountability	9 (52.9)	8 (47.1)			
ICS						
ICS1	Patient communication		1 (5.9)		15 (88.2)	1 (5.9)
ICS 2	Team management				16 (94.1)	1 (5.9)

PC, patient care; *SBP*, systems-based practice; PROF, professionalism; *MK,* medical knowledge; *PBLI*, practice-based performance improvement; *ICS*, patient-centered communication.
