# Supplementary material for: Promoting Achievement of Level 1 Milestones for Medical Students Going into Emergency Medicine
Source: West J Emerg Med. 2016 Dec 5;18(1):20–5. doi: 10.5811/westjem.2016.10.31247 (PMC5226757; doi:10.5811/westjem.2016.10.31247)
Supplement: Supplementary file 1 [file wjem-18-20-s001.docx]

Appendix.

Emergency Medicine Assessment Shift Checklist

Student: ____________________ Date: _________________________

|  |  | Yes | No | N/A |
| --- | --- | --- | --- | --- |
| 1. | Emergency stabilization |  |  |  |
|  | a. Did the student recognize abnormal vital signs? |  |  |  |
|  | b. Did the student recognize an unstable patient requiring immediate intervention? |  |  |  |
|  | c. Did the student perform a primary assessment on a critically ill or injured patient? |  |  |  |
|  | d. In the evaluation of an unstable patient, was the student able to discern relevant data to formulate a diagnostic impression and plan? |  |  |  |
| 2. | Performance of focused H&P examination |  |  |  |
|  | a. Did the student perform a comprehensive history and physical exam? |  |  |  |
|  | b. Did the student perform a focused history and physical exam which effectively addressed the chief complaint and urgent patient issues? |  |  |  |
|  | c. Was the student able to reliably communicate the history and physical exam (to the resident)? |  |  |  |
|  | d. Did the student ask patient for drug allergies? |  |  |  |
| 3. | Diagnosis |  |  |  |
|  | a. Did the student construct a list of potential diagnoses based on chief complaint and initial assessment? |  |  |  |
|  | b. Was the differential diagnosis prioritized based on the greatest likelihood of occurrence? |  |  |  |
|  | c. Did the differential diagnoses include emergent diagnoses with the greatest potential for morbidity or mortality? |  |  |  |
| 4. | Diagnostic studies |  |  |  |
|  | a. Did the student determine the necessity (or lack thereof) of diagnostic studies? |  |  |  |
|  | b. Did the student order appropriate diagnostic studies? |  |  |  |
|  | c. Did the student perform appropriate bedside diagnostic studies and procedures? |  |  |  |
| 5. | Pharmacotherapy |  |  |  |
|  | a. Did the student know the different classifications of pharmacologic agents and their mechanism of action? |  |  |  |
|  | b. Did the student apply medical knowledge for selection of appropriate agent for therapeutic intervention? |  |  |  |
|  | c. Did the student consider potential adverse effects of pharmacotherapy? |  |  |  |
| 6. | Observation and reassessment |  |  |  |
|  | a. Did the student recognize the need for patient re-evaluation? |  |  |  |
|  | b. Did the student ensure that necessary therapeutic interventions were performed during a patient’s ED stay? |  |  |  |
| 7. | Disposition |  |  |  |
|  | a. Did the student describe basic resources available for care of the emergency department patient? |  |  |  |
|  | b. Did the student formulate a specific follow-up plan with appropriate resource utilization? |  |  |  |
| 8. | Multitasking |  |  |  |
|  | a. Was the student able to manage a single patient amidst distractions? |  |  |  |
| 9. | Technology |  |  |  |
|  | a. Did the student use EPIC to order tests, medications and document notes? |  |  |  |
|  | b. Did the student review medications for patients in EPIC? |  |  |  |
| 10. | Professional values |  |  |  |
|  | a. Did the student demonstrate behavior that conveys caring, honesty, genuine interest and tolerance when interacting with a diverse population of patients and families? |  |  |  |
|  | b. Did the student demonstrate compassion, integrity, respect, sensitivity and responsiveness? |  |  |  |
| 11. | Accountability |  |  |  |
|  | a. Was the student on time, appropriately dressed, and ready to work? |  |  |  |
|  | b. Did the student maintain patient confidentially? |  |  |  |
|  | c. Did the student consistently recognize their own limits and asks for assistance when needed? |  |  |  |
| 12. | Patient centered communication |  |  |  |
|  | a. Did the student establish rapport with and demonstrate empathy toward patients and their families? |  |  |  |
|  | b. Did the student listen effectively to patients and their families? |  |  |  |
|  | c. Did the student elicit the patient’s reasons for seeking health care? |  |  |  |
|  | d. Did the student elicit the patient’s expectations from the ED visit? |  |  |  |
| 13. | Team management |  |  |  |
|  | a. Did the student participate as a member of a patient care team? |  |  |  |
|  | b. Did the student communicate pertinent information to emergency physicians and other healthcare colleagues? |  |  |  |
